# Gold Nanoparticles for Targeting Varlitinib to Human Pancreatic Cancer Cells

**DOI:** 10.3390/pharmaceutics10030091

**Published:** 2018-07-12

**Authors:** Sílvia Castro Coelho, Daniel Pires Reis, Maria Carmo Pereira, Manuel A. N. Coelho

**Affiliations:** LEPABE—Laboratory for Process Engineering, Environment, Biotechnology and Energy, Faculty of Engineering, University of Porto, Rua Dr Roberto Frias, 4200-465 Porto, Portugal; reis.danielp@gmail.com (D.P.R.); mcsp@fe.up.pt (M.C.P.); mcoelho@fe.up.pt (M.A.N.C.)

**Keywords:** gold nanoparticles, tyrosine conjugation, pancreatic cells

## Abstract

Colloidal gold nanoparticles are targeting probes to improve varlitinib delivery into cancer cells. The nanoconjugates were designed by the bioconjugation of pegylated gold nanoparticles with varlitinib via carbodiimide-mediated cross-linking and characterized by infrared and X-ray photoelectron spectroscopy. The drug release response shows an initial delay and a complete drug release after 72 h is detected. In vitro experiments with MIA PaCa-2 cells corroborate that PEGAuNPsVarl conjugates increase the varlitinib toxic effect at very low concentrations (IC50 = 80 nM) if compared with varlitinib alone (IC50 = 259 nM). Our results acknowledge a decrease of drug side effects in normal cells and an enhancement of drug efficacy against to the pancreatic cancer cells reported.

## 1. Introduction

Varlitinib is a tyrosine kinase inhibitor of the epidermal growth factor receptor (EGFR) family, controlling cell growth, differentiation, and survival. It selectively and reversibly binds to both EGFR (ErbB-1) and Her-2/neu (ErbB-2) and prevents their phosphorylation and activation [1]. Several reports suggested varlitinib as a selective anticancer-drug and inhibitor of EGFR/ErbB-2 [1,2,3]. The tyrosine kinase inhibitor can reverse, significantly, the multidrug resistance (MDR) in cancer cells resulting from the inhibition of the ATP-binding cassette (ABC) transporters that act in extruding a variety of chemotherapeutic agents out of the tumour cells [2]. Some studies reported an efficient in vitro activity of varlitinib in combination with other anticancer drugs in several tumour models, suggesting varlitinib not only as a potent single tyrosine kinase inhibitor but also with high tolerability with other drugs [2,4].

The primary problem in the cancer treatments with chemotherapeutic agents has been the high toxicity and low bioavailability of the anticancer therapy. The tumour heterogeneity and the MDR are the key challenge in anticancer therapy [5]. Trying to avoid such problems, nanoparticles (NPs) have been a challenge for delivering of the anticancer drugs to the tumour cells [6]. They have been promising tools to attain better retention and release of therapeutic and diagnosis agents, and furthermore, to overcome the conventional therapeutic limitations [7,8,9]. A good effort of this application are inorganic nanosized vehicles such as gold nanoparticles (AuNPs) [10]. Due to their distinct optical and chemical properties—easy preparation, characteristic surface plasmon resonance (SPR) band, simple chemistry, and high functionalizable surface—they have been studied as drug delivery vehicles and imaging agents [11,12,13]. They present a significant biocompatibility and their production costs are very low, which facilitated their use [14,15]. AuNPs can be synthesized via different methods, with different shape (spheres, rods, tubes, wires, ribbons, cubic, hexagonal, triangular) and size [16,17,18]. AuNPs present small sizes that can allow the enhanced permeation and retention (EPR) effect and minimize reticuloendothelial system (RES) clearance [19,20]. There are successful in vitro studies reporting a better inhibition of tumour cell proliferation using conjugated gold nanoparticles with anticancer drugs compared with the same free drugs [21,22,23,24,25]. Aryal et al. reported the conjugation of AuNPs with doxorubicin using thiolated methoxy polyethylene glycol as a linker [13]. Coelho et al. studied pegylated AuNPs with afatinib, which present a potential drug delivery nanosystem to enhance the toxicity of the drug against pancreatic as well as non-small lung cancer cell lines [25]. To improve the stability of the colloidal suspension and to inhibit protein adsorption to their surface, the nanoparticles can be modified covalently [15]. α-thiol-carboxyl (polyethylene glycol) can be bound to the surface of gold nanoparticles [15]. This uncharged polymer is non-toxic and minimizes the electrostatic interactions with plasma proteins [26,27]. The coupling reaction of the activator *N*-ethyl-*N*′-(3-dimetylaminopropyl) carbodiimide (EDC) and sulfo-*N*-hydroxysuccinimide (NHSS) is used to mediate the formation of linkage between carboxylic and amino-terminated groups [28].

In the present study, the aim was to obtain conjugated gold nanoparticles to evaluate its effect into human pancreatic cells: MIA PaCa-2, a pancreatic cancer cell line that express high levels of HER2/neu and EGFR [21,29,30], and hTERT-HPNE, an immortalized human pancreatic duct epithelial cells [31]. Conjugates of gold nanoparticles to varlitinib have not yet been reported.

Pegylated gold nanoparticles were synthesized and conjugated with varlitinib via carbodiimide-mediated cross-linking. The nanoconjugate was characterized by using ultraviolet visible (UV-Vis), dynamic light scattering (DLS) and laser Doppler velocimetry, Attenuated Total Reflectance-Fourier Transform Infrared Spectroscopy (ATR-FTIR), X-ray photoelectron spectroscopy (XPS), transmission electron microscopy (TEM) techniques, in vitro drug release, and in vitro drug stability analysis. Our results showed that pegylated gold nanoparticles represent a promising drug delivery nanosystem, enhancing the varlitinib cell toxicity in pancreatic cancer cell lines.

## 2. Materials and Methods

### 2.1. Materials

Varlitinib was acquired from Selleck Chemicals LLC (Houston, TX, USA). Phosphate buffered saline (PBS) and fetal bovine serum (FBS) was purchased from Invitrogen Co. (Scotland, UK). Dimethyl sulfoxide (DMSO), trisodium citrate dihydrate and tetrachloroauric (III) acid—HAuCl_4_; 99.99% trace metals basis, 30 wt % in dilute HCl—were acquired from Sigma-Aldrich Química (Sintra, Portugal). a-thiol-w-carboxyl (polyethylene glycol) (HS-C11-(EG)3-OCH2-COOH; molecular weight 394.57 Da) was obtained from Prochimia (Gdynia, Poland).

### 2.2. Cell Culture

Immortalized human pancreatic duct epithelial cells (hTERT-HPNE) were provided by Professor M. A. Hollingsworth (UNNC—Omaha, NE, USA). Human pancreatic carcinoma cells (MIA PaCa-2) were obtained from the LGC Standards (Barcelona, Spain). The cells were maintained in DMEM medium, supplemented with 10% FBS under 5% CO_2_ humidified atmosphere at 37 °C.

### 2.3. Synthesis of Pegylated Gold Nanoparticles

Gold nanoparticles (AuNPs) were prepared by the reduction process of HAuCl_4_ through a solution of trisodium citrate [17,32]. Then, AuNPs were functionalized with a-thiol-w-carboxyl (polyethylene glycol) layer (molar ratio 1:1000, respectively)—PEG. PEGAuNPs were collected by centrifugation (13,400 g, 10 min) and resuspended in ultrapure water. The concentration of the PEGAuNPs, determined by the Lambert-Beer Law was 15.08 nM.

### 2.4. Conjugation of Varlitinib to PEGAuNP, PEGAUNPsVarl

Varlitinib was conjugated to PEGAuNPs using the EDC/NHSS coupling (molar ratio 1000:1, respectively) for 2.5 h. The PEGAuNPVarl was centrifuged (13,000× *g*) to remove the unbound varlitinib drug.

### 2.5. Dynamic Light Scattering and Electrophoretic Mobility Measurements

Size distribution and zeta potential of nanoconjugates were determined by dynamic light scattering and laser doppler velocimetry, respectively, using a Zetasizer Nano ZS (Malvern Instruments Ltd., Malvern, UK), at 25 °C.

### 2.6. Ultraviolet Visible Spectroscopy

PEGAuNPs and PEGAuNPsVarl were analysed by UV-Vis spectroscopy (Shimadzu UV-1700 PharmaSpec spectrophotometer, Kyoto, Japan), using a 1 cm quartz cuvette, at room temperature.

### 2.7. Attenuated Total Reflectance-Fourier Transform Infrared Spectroscopy (ATR-FTIR)

The suspensions of PEGAuNPsVarl and PEGAuNPs, and varlitinib solutions were analysed by ATR-FTIR spectroscopy (ALPHA FTIR Spectrometer, Bruker, Billerica, MA, USA). Spectral scanning was acquired in the 4000–400 cm^−1^, resolution of 4 cm^−1^, and 64 scans, at 25 °C.

### 2.8. Transmission Electron Microscopy (TEM) Analysis

TEM images were acquired using a Jeol JEM-1400 (Peabody, MA, USA), JEOL operated at 60 kV. An amount of 5 μL of each sample was placed on carbon formvar-coated grid and let to adsorb for 5 min. After, the grid was washed twice with deionized (DI) water to remove the excess.

### 2.9. X-Ray Photoelectron Spectroscopy (XPS) Analysis

XPS was performed on a Kratods Axis Ultra HAS instrument (Manchester, UK) using a monochromator Al X-ray anode source operated at 90 W. Samples—AuNPs, PEGAuNPs, and PEGAuNPsVarl—were prepared by drop on a clean microscope slide and the drops were allowed to air dry before the analysis.

### 2.10. Varlitinib/PEGAuNPs Conjugation Efficiency

The PEGAuNPsVarl formulations were centrifuged (13,000× *g*, 15 min) and the supernatant was collected to measure varlitinib concentration by fluorescence measures (excitation at 360 nm, emission at 485 nm). The conjugation efficiency was evaluated by:

(Varlitinib initial concentration − Varlitinib supernatant concentration)/Varlitinib initial concentration

The results are presented as mean and SD of at least three independent experiments.

### 2.11. In Vitro Drug Release Studies

The in vitro release profile of PEGAuNPsVarl was performed by dialysis. Nanoconjugates with 4.2 μM of varlitinib concentration were incubated in PBS 0.01 M, pH 7.4, at 37 °C with constant magnetic stirring in regenerated cellulose. Varlitinib concentration of the dialysate buffer was determined with time through fluorescence measures using a microplate reader (PowerWave HT Microplate Spectrophotometer, BioTek Instruments Inc., Winooski, VT, USA) (excitation at 360 nm, emission at 485 nm). Varlitinib concentration of the dialysate buffer was determined with time through fluorescence measures using a microplate reader (PowerWave HT Microplate Spectrophotometer, BioTek, Instruments Inc., Winooski, VT, USA) (excitation at 360 nm, emission at 485 nm).

### 2.12. In Vitro Stability Studies

PEGAuNPs 8 nM and PEGAuNPsVarl 5 nM were incubated in PBS 0.01 M, at 4 °C and in FBS 10% (*v/v*) in PBS solution, at 37 °C. Samples were evaluated at several time points post incubation during 72 h by using DLS, UV-Vis spectroscopy and laser Doppler velocimetry.

### 2.13. In Vitro Cytotoxicity Study

In vitro cytotoxicity of varlitinib and PEGAuNPsVarl against pancreatic cell lines was evaluated by SRB (colorimetric) [33]. Briefly, the MIA PaCa-2 and hTERT- HPNE cells were seeded on 96-well plates with a cell density at 1000 cells per well, under normal conditions (5% CO_2_ humidified atmosphere at 37 °C) and allowed to adhere for 24 h. Then, the cells were treated for 48 h with varlitinib and PEGAuNPsVarl at the concentrations ranging between 10 and 1000 nM varlitinib. Cells were fixed with 10% (*w/v*) TCA for 60 min on ice. Next, the cells were washed with water and stained with 50 μL of SRB solution. The unbound dye was removed by washing with 1% (*v/v*) acetic acid. The dried cells and the protein-bound stain were solubilized with 10 mM Tris solution. The SRB absorbance was measured at 560 nm in a microplate reader (Synergy HT Multi-Mode Microplate Reader, BioTek Instruments Inc., Winooski, VT, USA). The IC50 (concentration for 50% of cell survival) and GI50 (50% of growth inhibition) values were determined. The absorbance of the wells containing the NPs or drug and the absorbance of the wells containing untreated cells following a 48-h incubation period were subsequently compared with that of the wells containing the cells that have been fixed at time zero (corresponding to incubation of the nanoparticles and drug).

### 2.14. Statistical Analysis

Values are reported as mean of three independent experiments. Student’s *t*-test statistical analysis was used to determine statistical significance ((*p* < 0.05).

## 3. Results and Discussion

**Figure 1:**
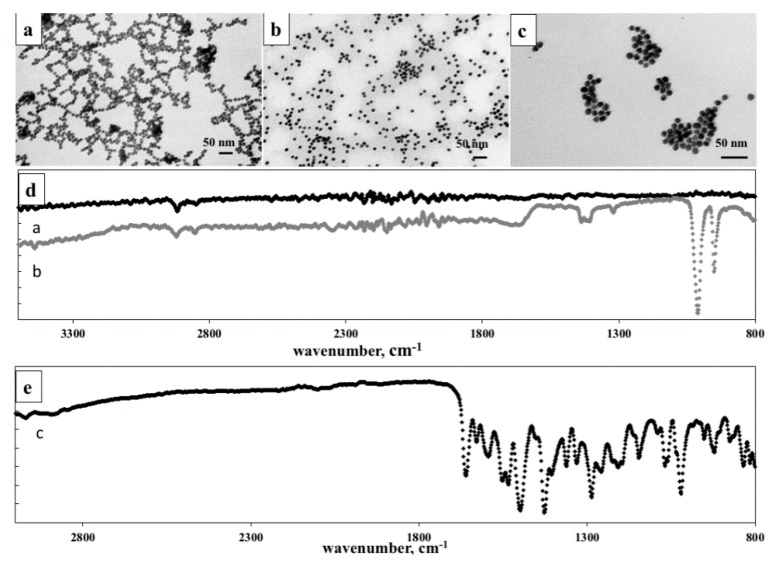
TEM images of (**a**) AuNPs, (**b**) PEGAuNPs, (**c**) PEGAuNPsVarl. Scale bar is 50 nm; (**d**) FTIR spectra of PEGAuNPsVarl (black line) and PEGAuNPs (grey line), (**e**) FTIR spectra of varlitinib (black dots). The spectra were shifted for a better visualization.

Spherical AuNPs were firstly synthesized by the classical Turkevitch method and presented in TEM image (Figure 1a) [32,33]. The nanoparticles were prepared by reduction of the HAuCl_4_ solution with sodium citrate. They present a hydrodynamic diameter of 20.0 ± 0.2 nm (Table 1), results obtained by DLS analysis.

The final concentration of stock AuNPs was 14 ± 1 nM, determined by Lambert–Beer law and absorbance peak at 520 nm characteristic by the reduction of HAuCl_4_ to AuNPs [34].

The morphology of different AuNPs samples was characterized by TEM. After functionalization of the AuNPs surface with PEG layer via Au-S bonds, PEGAuNPs did not change in shape and the size is increased slightly (Figure 1b). This result agrees with the size distribution (PdI 0.3) indicating a good monodisperse distribution of the colloidal suspension which nanoparticles have an average hydrodynamic diameter of 27 ± 2 nm and a zeta potential −34 ± 1 mV confirming their stability (Table 1). The nanoparticles were stable for several months when stored at 4 °C in aqueous dispersion. The concentration of PEGAuNPs 8.9 ± 0.8 nM was estimated from UV-Vis spectra. As shown in Figure S1 UV-Vis spectra showed the presence of a surface plasmon resonance band centered at 522 nm, determined by UV-Vis absorption spectroscopy.

**Figure 2:**
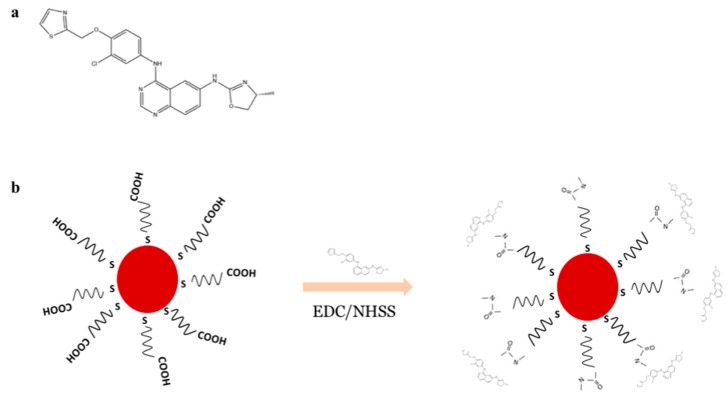
(**a**) Chemical structure of varlitinib; (**b**) Scheme of nanoconjugate PEGAuNPsVarl preparation.

The resultant nanoparticles—PEGAuNPs—were conjugated with varlitinib (PEGAuNPsVarl) by using the EDC/NHSS crosslinking of carboxylic acids from PEGAuNPs with secondary amine group of varlitinib (Figure 2a), illustrated in Figure 2b.

Table 1 shows the average hydrodynamic diameter measurements of PEGAuNPsVarl. They have 28 ± 2 nm and the zeta potential is −33 ± 1 mV. Also, TEM image (Figure 1c) illustrated well-defined nanoconjugates with small diameters as DLS measurements and the formation of some aggregated nanoparticles. The surface plasmon resonance peak of the designed nanoconjugates showed a red shift of 2 nm compared to that of original PEGAuNPs (wavelength of 522 nm) and their estimated concentration was 3.5 ± 0.8 nM.

The varlitinib conjugation efficiency was determined through fluorescence analysis. Per the data, 84 ± 1% (*w/w*) of varlitinib was conjugated with PEGAuNPs (by subtracting the unbound varlitinib in the supernatant solution). Therefore, the final varlitinib concentration in stock PEGAuNPs solution was determined to be 4.4 ± 0.5 µM.

Figure 1d indicated the FTIR analysis of nanoparticles to understand and confirm the covalent bonds. In Figure 1d, the ATR-FTIR spectrum of unmodified PEGAuNPs showed characteristic peaks at 1741 cm^−1^ from carbonyl C=O stretching and at 1317 cm^−1^ from C–OH stretching group of the ethylene glycol monomers. At 1151 and 1165 cm^−1^, the peaks to the C–O–C groups were observed, and at 2917 cm^−1^ it appeared the vibrational stretches of –CH_2_ groups of long alkane chains from PEG. In the FTIR spectrum of PEGAuNPsVarl, the peak at 1671 cm^−1^ indicates the C=N that can be assigned to the imine vibration from reaction of secondary amine of varlitinib with carboxylic acid of PEGAuNPs (Figure 1d) [28]. The peaks at 1407, 1418, and 1437 cm^−1^ represent the C=C stretch from aromatic groups of varlitinib (Figure 1e). At 807 cm^−1^, the peaks are visible of C–H aromatic out-of-plane bending. At 950 and 1011 cm^−1^, varlitinib peaks appeared –C–H aromatics out-of-plane bend and C–N amine group, respectively.

**Figure 3:**
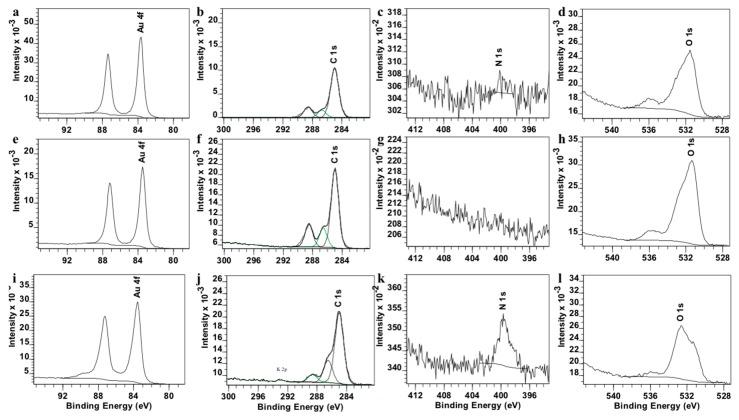
XPS deconvolution of Au 4f (**a**,**e**,**i**), C1s (**b**,**f**,**j**), N (**c**,**g**,**k**), and O (**d**,**h**,**l**) of AuNPs (**a**–**d**), PEGAuNPs (**e**–**h**), and PEGAuNPsVarl (**i**–**l**).

Moreover, AuNPs, PEGAuNPs, and PEGAuNPsVarl were further analysed by XPS as shown in Figure S2. The contributions of elements, Au, C, N, O atoms are displayed in Figure 3. The binding energy of Au 4f of samples exhibits at 83.6 and 87.25 eV, which is higher than that of PEGAuNPs at 83.54 and 87.18 eV. Also, these data show the presence of three carbon peaks at 284.9, 286.7, and 288.9 indicating sp^3^—(in saturated hydrocarbons) and sp^2^—hybridized carbons (such as C=C and C=O). It corroborates with a covalent interaction between AuNPs and PEG-COOH. The signal of N at 399.76 eV is observed for PEGAuNPsVarl (Figure S2c) and the signal of O decreased, suggesting a covalent bonding of the varlitinib nitrogen to PEGAuNPs, in accordance with FTIR data.

Table 2 showed the XPS elemental composition on the regions of interest. According to the XPS composition data, the signal of C increased from 60.4% (AuNPs) of the sample to 65.1% (PEGAuNPs and PEGAuNPsVarl), representing the good functionalization with PEG layer. Also, the signal of N on PEGAuNPsVarl is distinct (1.60%), indicating the presence of varlitinib.

The time-dependent absorbance spectra, hydrodynamic diameter, and zeta potential were performed to investigate the stability of PEGAuNPsVarl and PEGAuNPs in PBS at 4 °C for 72 h, and were presented in Figures S3a–S5a. PEGAuNPs were stable over 72 h of incubation in PBS at 4 °C. They had 29.8 nm and a zeta potential of −24.0 ± 0.7 mV (Figures S3a and S4a). The behaviour of PEGAuNPsVarl in PBS at 37 °C did not change significantly. In fact, the particles changed its hydrodynamic diameter to 31.3 nm (Figure S3a), data in accordance with the increase of the wavelength value of the plasmon peak (Figure S5a). Also, on Figure S4a, it was observed that NPs had −24.1 ± 0.5 mV of zeta potential which remained unchanged for 48 h; after this period, it tends to be less negative (−22.4 ± 0.4 mV).

The PEGAuNPsVarl stability in the presence of fetal bovine serum (FBS) was evaluated by hydrodynamic diameter, zeta potential measurements and time-dependent absorbance spectra, at 37 °C for 72 h (Figures S3b–S5b). In the presence of FBS, two populations are present: a core population with 33 ± 2 nm (86%) and a minor population with 134 ± 4 nm (14%), due to the FBS protein absorption into the nanoconjugates (Figure S3b). A slight increase was observed at 72 h. The PEGAuNPs zeta potential values decreased to −9 ± 1 mV, which are justified by the adsorption of proteins and ions to the nanoconjugates reducing the electrostatic repulsion between them favouring some aggregation.

**Figure 4:**
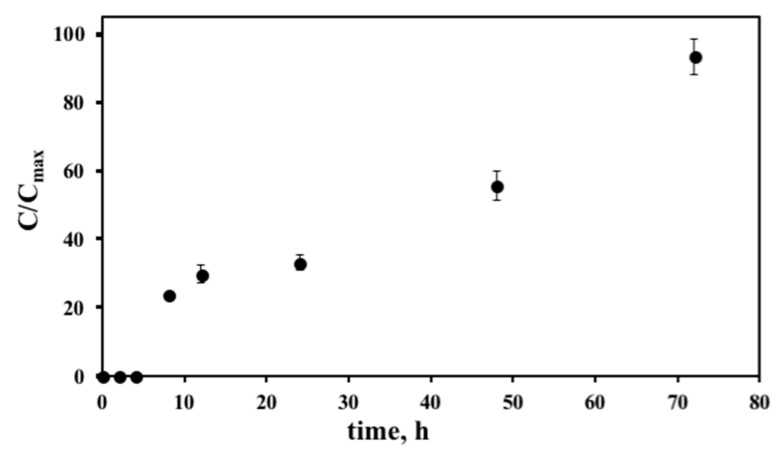
The varlitinib release profile of the PEGAuNPsVarl in PBS at pH 7.4 and 37 °C.

The in vitro drug controlled release experimental of PEGAuNPsVarl was performed in PBS (0.01 M, pH 7.4 at 37 °C) through a regenerated cellulose dialysis membrane with an initial varlitinib concentration in NPs of 4.2 μM. Figure 4 presented the drug release data. It is possible to visualize an initial delay of 4 h. After 8 h, around 20% of the varlitinib amount was released. Figure 4 indicates a slow and controlled release of the drug conjugated with the nanoparticles that might be explained from conjugated NPs. The conjugated PEGAuNPs release about 93 ± 6% of the varlitinib for 72 h, suggesting the disruption of the covalent bond of thiol-PEG with gold nanoparticles due to the temperature increase [35].

*C*_max_ corresponds to the total amount of the drug added. Results are shown as mean ± SEM of three independent experiments.

**Figure 5:**
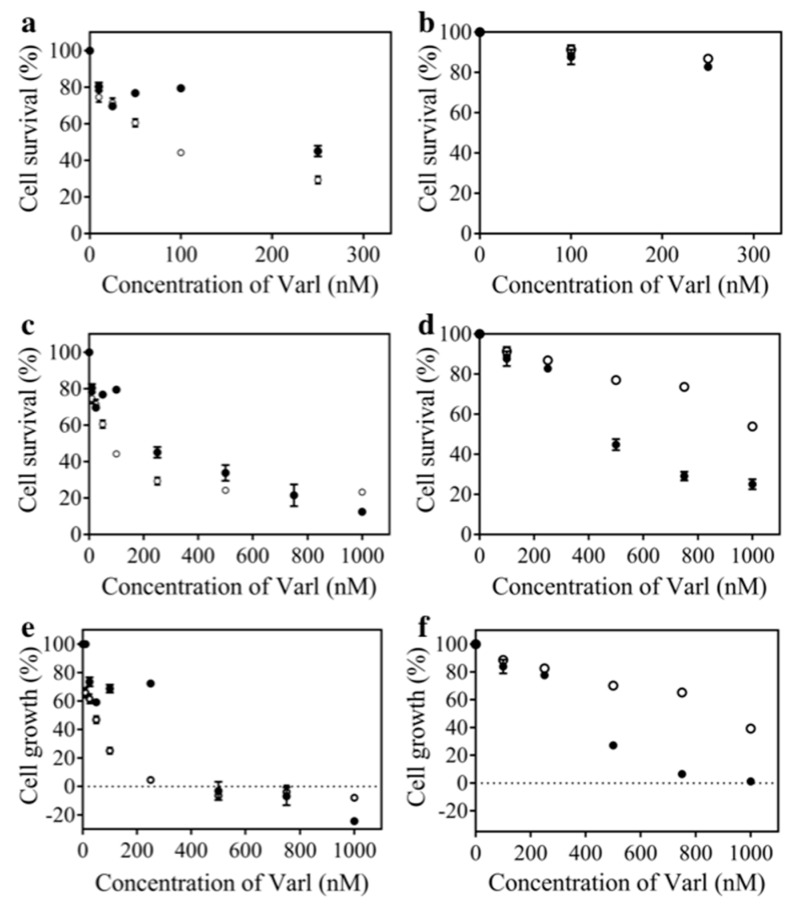
Cytotoxic effects of PEGAuNPsVarl (◯) and varlitinib alone (●) after 48 h a treatment on the cell survival (varlitinib concentration range from 10 to 300 nM) (**a**,**b**); cell survival (varlitinib concentration range from 10 to 1000 nM) (**c**,**d**) and on cell growth (**e**,**f**) of MIA PaCa-2 (**a**,**c**,**e**) and hTERT-HPNE (**b**,**d**,**f**), determined by a SRB assay.

The in vitro cytotoxic effects after treatment with varlitinib alone and PEGAuNPsVarl were assessed on MIA PaCa-2 and hTERT-HPNE cell. Treatment with PEGAuNPs at concentrations up to 2 nM, during 48 h of incubation, did not presented effect on the cell growth (data not shown) corroborating non-toxicity of the PEGAuNPs [33]. The effect of varlitinib at different concentrations (10 to 1000 nM) was tested and cell growth analysed. Figure 5 shows the cell survival results of the cell lines after incubation with PEGAuNPsVarl and varlitinib alone for 48 h, and PEGAuNPsVarl toxicity was compared with varlitinib alone. The cell survival of MIA PaCa-2 cells decreases after exposure with both free and conjugated varlitinib (Figure 5a,c). On MIA PaCa-2, for varlitinib concentration of 100 nm, toxicity of varlitinib conjugated PEGAuNPs was higher than varlitinib alone (44% of the cell survival for VarlPEGAuNPs and 80% for varlitinib alone). These results can be explained by cancer cell environment specifically acidic pH gradient and hypoxia promoting nanoparticle uptake via endocytosis and, as a result, drug concentration increases in the cytoplasm [33,36,37,38]. Also, MIA PaCa-2 cells overexpress high levels of HER2/neu and EGFR [21,29,30] that can be inhibited and reversibly bounded to varlitinib [1].

The same trend is observed when analyzing inhibition of cell growth in response to varlitinib alone and conjugated to PEGAuNPs. For MIA PaCa-2s, the nanoconjugate improves the varlitinib activity resulting lower GI50 values (Table 3 and Figure 5e). In 48 h of incubation, varlitinib alone inhibits the MIA PaCa-2 cell growth by 50% with 259.1 ± 0.4 nM of concentration which is higher when compared with 80 ± 4 nM of varlitinib concentration conjugated with PEGAuNPs. The efficacy of the PEGAuNPsVarl to induce cell death is more pronounced than that of varlitinib alone for varlitinib concentrations above 250 nM (Figure 5).

**Figure 6:**
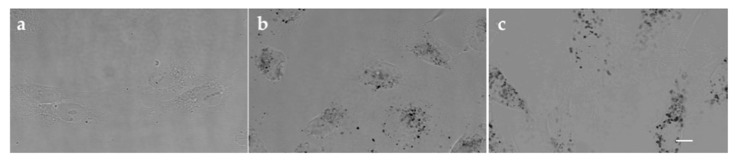
Confocal reflectance images of the hTERT-HPNE cells after 48 h incubation: (**a**) control untreated cells; (**b**) 0.5 nM of PEGAuNPs; (**c**) 0.5 nM of PEGAuNPsVarl. Scale bar in all images is 10 μM.

For the hTERT-HPNE cells, the same effect is not observed (Figure 5b,d). Our data show that PEGAuNPsVarl inhibited about 23% of cell survival for varlitinib concentration of 500 nM. For the same concentration, varlitinib alone inhibited more than two times (cell survival is around 55%). hTERT-HPNE displayed higher sensitivity and they presented a significant higher inhibition to varlitinib alone than in presence of the nanoconjugate. In addition, the varlitinib concentrations of PEGAuNPsVarl and varlitinib alone inhibiting cell survival in 50% (IC50 values) are 1186 ± 4 μM and 478 ± 5 μM, respectively (Table 3). This effect might be due to the protection of varlitinib by PEGAuNPs. The nanoparticle behaviour in hTERT-HPNE cells could be related with pH gradient. Ding et al. reported that in normal cells, the neutral pH gradient does not promote the nanoparticle internalization when compared with the cancer cell acidic conditions [39]. It was observed a small number of nanoparticles in hTERT-HPNE cytoplasm (Figure 6) in contrast with the nanoparticle concentration detected in pancreatic cancer cells (S2-013) with a clear PEGAuNP accumulation near the nucleus [7,33]. In cancer cells, we have realized a stochastic dynamic formation of endosomes with several gold nanoparticles with a high electron density. This particularity was not observed in hTERT-HPNE cells. The hTERT-HPNE cell morphology does not change significantly with the incubation of the PEGAuNPs alone and conjugated with drug due to the low nanoparticle internalization by the cells, as observed in both tests (Figure 6b,c). The new findings of PEGAuNPsVarl effect on MIA PaCa-2 and hTERT-HPNE cells corroborate the mechanisms proposed and reported by Coelho et al. [33]

An evan of inhibition hTERT-HPNE cell growth in response to PEGAuNPsVarl and varlitinib alone is observed on Figure 5f). For hTERT-HPNE, the GI50 concentration required to inhibit is 2.5 times lower to varlitinib conjugated with PEGAuNPs than varlit alone (916 ± 3 nM and 354 ± 5 nM, respectively). By other hand, the analysis of the balance between cell proliferation and cell death of hTERT-HPNE only showed a decrease of the inhibitory growth with time revealing cell inhibition for both treatments.

## 4. Conclusions

In summary, a well-defined varlitinib delivery system PEGAuNPsVarl was successfully designed and prepared through the EDC/NHSS coupling reaction with a conjugation efficiency of 84%*.* The in vitro release profiles show a delay on varlitinib release due to the coupling process. The PEGAuNPsVarl shows a significant cancer cell survival inhibition for MIA PaCa-2 cells. In fact, cell survival appeared to decrease by 22–80% after PEGAuNPsVarl treatment with varlitinib concentration in range from 10 to 1000 nM, if compared to varlitinib alone. In contrast, cell inhibition in hTERT-HPNE cells by PEGAuNPsVarl is lower, denoting a drop of the nanoconjugate toxic effects in non tumour cells. The varlitinib therapeutic effect is enhanced by the controlled release of the anticancer drug after conjugation with functionalized gold nanoparticles. Our findings indicate that PEGAuNPs can be used as an effective vehicle for varlitinib inhibitor.

The drug delivery system shows potential antineoplastic activity for the treatment of EGFR overexpressed family, decreasing drug doses and the multi-drug resistance effects.

## Figures and Tables

**Table 1:** Physical characteristics of AuNPs, PEGAuNPs, and PEGAuNPsVarl.

Physical Characteristics	AuNPs	PEGAuNPs	PEGAuNPsVarl
size, nm	20.0 ± 0.2	27 ± 2	28 ± 2
polydispersity index	0.2	0.3	0.7
zeta potential, mV	−37 ± 3	−34 ± 1	−33 ± 1

AuNPs: gold nanoparticles; PEGAuNPs: Pegylated gold nanoparticles; PEGAuNPsVarl: Pegylated gold nanoparticles conjugated with varlitinib.

**Table 2:** XPS elemental composition of AuNPs, PEGAuNPS, and PEGAuNPsVarl (at % normalized to 100%).

Element	AuNPs	PEGAuNPs	PEGAuNPsVarl
C 1s	60.44	65.05	65.06
N 1s	0.06	-	1.60
Au 4f	16.77	4.82	13.80
O 1s	22.73	30.14	19.55

**Table 3:** Half maximal inhibitory concentration (IC50) and effect of varlitinib alone and PEGAuNPsVarl on the growth inhibition (GI50) on the pancreatic cell lines—MIA PaCa-2 and hTERT-HPNE.

Parametric Analysis	MIA PaCa-2		hTERT-HPNE	
	PEGAuNPsVarl	varlitinib	PEGAuNPsVarl	varlitinib
IC50 (nM)	80 ± 4	259.1 ± 0.4	1186 ± 4	478 ± 5
GI50 (nM)	40 ± 1	268 ± 7	916 ± 3	354 ± 5

## Supplementary Materials

The following are available online at http://www.mdpi.com/1999-4923/10/3/91/s1, Figure S1. UV-Vis spectra of AuNPs, PEGAuNPs and PEGAuNPsVarl. Figure S2. XPS survey spectra of AuNPs (a), PEGAuNPs (b) and PEGAuNPsVarl (c). Figure S3. Size distribution analysis of PEGAuNPs (black column) and PEGAuNPsVarl (striped column) in (a) PBS 0.01 M at 4 °C; (b) FBS at 37 °C, after incubation for different periods of time. Figure S4. Stability analysis of zeta potential property of PEGAuNPs (▲) and PEGAuNPsVarl (●) in (a) PBS 0.01 M at 4 °C; (b) FBS at 37 °C, after incubation for different periods of time. Figure S5. UV-Vis spectra of PEGAuNPs (▲) and PEGAuNPsVarl (●) in (a) PBS 0.01 M at 4 °C; (b) FBS at 37 °C, after incubation for different periods of time.
