# Supplementary material for: Gold Nanoparticles for Targeting Varlitinib to Human Pancreatic Cancer Cells
Source: Pharmaceutics. 2018 Jul 12;10(3):91. doi: 10.3390/pharmaceutics10030091 (PMC6161021; doi:10.3390/pharmaceutics10030091)
Supplement: Supplementary file 1 [file pharmaceutics-10-00091-s001.zip › pharmaceutics-306069-supplementary.docx]

Supplementary material for “Gold Nanoparticles
for Targeting Varlitinib to Human Pancreatic
Cancer Cells”

Sílvia Castro Coelho^*^, Daniel Pires Reis, Maria Carmo Pereira, Manuel A.N. Coelho


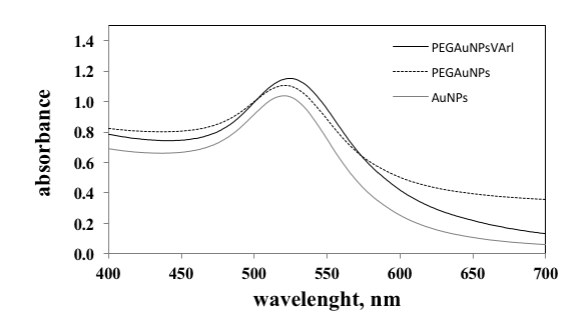


AuNPs: gold nanoparticles

PEGAuNPs: Pegylated gold nanoparticles

PEGAuNPsVarl: Pegylated gold nanoparticles conjugated with varlitinib

**Figure S1.** UV-Vis spectra of AuNPs, PEGAuNPs and PEGAuNPsVarl.


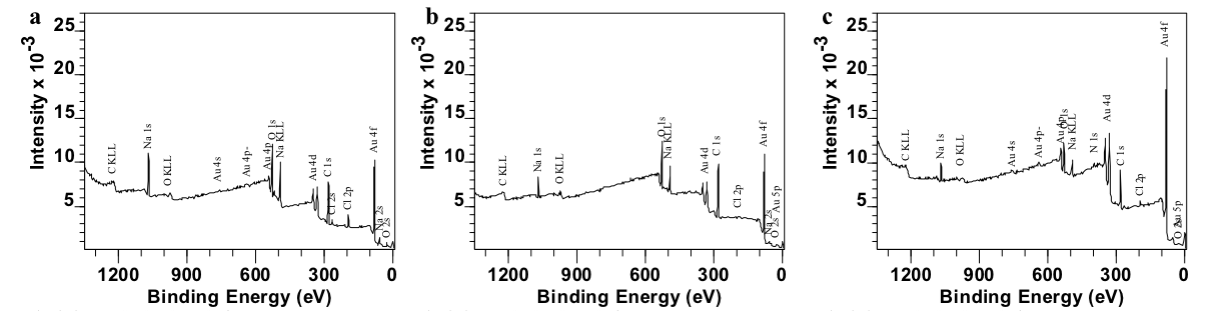


AuNPs: gold nanoparticles

PEGAuNPs: Pegylated gold nanoparticles

PEGAuNPsVarl: Pegylated gold nanoparticles conjugated with varlitinib

**Figure S2.** XPS survey spectra of AuNPs (**a**), PEGAuNPs (**b**) and PEGAuNPsVarl (**c**).


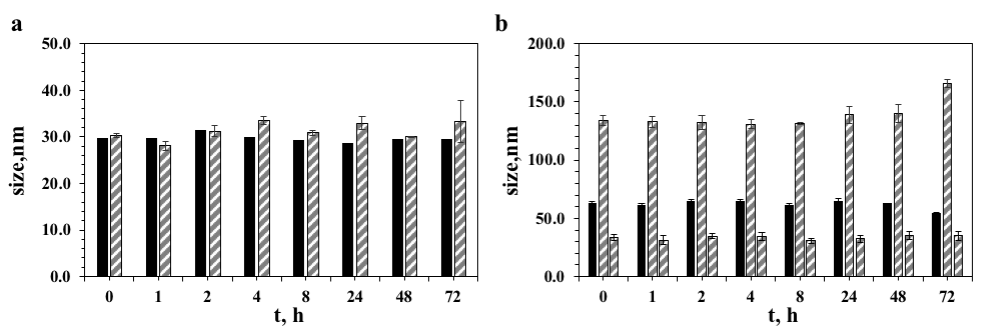


PEGAuNPs: Pegylated gold nanoparticles

PEGAuNPsVarl: Pegylated gold nanoparticles conjugated with varlitinib

**Figure S3.** Size distribution analysis of PEGAuNPs (black column) and PEGAuNPsVarl (striped column) in (a) PBS 0.01 M at 4 ºC; (b) FBS at 37 ºC, after incubation for different periods of time.


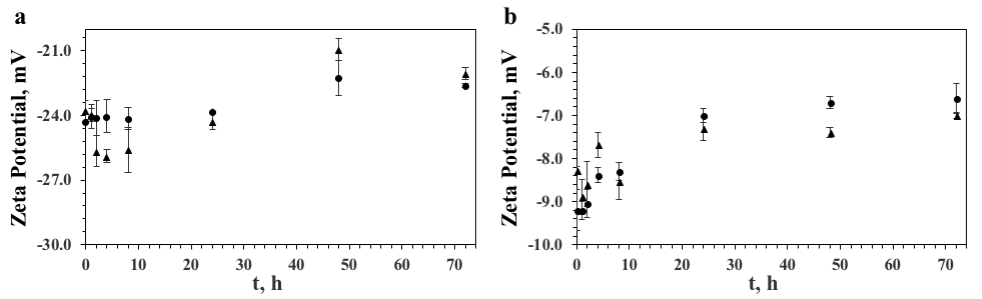


PEGAuNPs: Pegylated gold nanoparticles

PEGAuNPsVarl: Pegylated gold nanoparticles conjugated with varlitinib

**Figure S4.** Stability analysis of zeta potential property of PEGAuNPs (▲) and PEGAuNPsVarl (●) in (a) PBS 0.01 M at 4 ºC; (b) FBS at 37 ºC, after incubation for different periods of time.


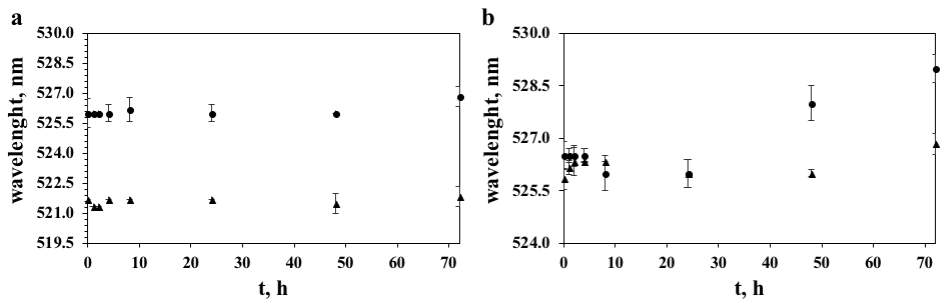


.

PEGAuNPs: Pegylated gold nanoparticles

PEGAuNPsVarl: Pegylated gold nanoparticles conjugated with varlitinib

**Figure S5.** UV-Vis spectra of PEGAuNPs (▲) and PEGAuNPsVarl (●) in (**a**) PBS 0.01 M at 4 ºC; (**b**) FBS at 37 ºC, after incubation for different periods of time.
